# The additive impact of cardio‐metabolic disorders and psychiatric illnesses on accelerated brain aging

**DOI:** 10.1002/hbm.25769

**Published:** 2022-02-03

**Authors:** Meghann C. Ryan, L. Elliot Hong, Kathryn S. Hatch, Si Gao, Shuo Chen, Krystl Haerian, Jingtao Wang, Eric L. Goldwaser, Xiaoming Du, Bhim M. Adhikari, Heather Bruce, Stephanie Hare, Mark D. Kvarta, Neda Jahanshad, Thomas E. Nichols, Paul M. Thompson, Peter Kochunov

**Affiliations:** ^1^ Maryland Psychiatric Research Center, Department of Psychiatry University of Maryland School of Medicine Baltimore Maryland USA; ^2^ Division of Biostatistics and Bioinformatics, Department of Public Health and Epidemiology University of Maryland School of Medicine Baltimore Maryland USA; ^3^ Department of Clinical Research and Leadership School of Medicine and Health Sciences, George Washington University Washington District of Columbia USA; ^4^ Department of Biostatistics School of Public Health, Cheeloo College of Medicine, Shandong University Jinan China; ^5^ Imaging Genetics Center, Stevens Neuroimaging & Informatics Institute, Keck School of Medicine of USC Los Angeles California USA; ^6^ Nuffield Department of Population Health of the University of Oxford Oxford UK

**Keywords:** accelerated brain aging, cardio‐metabolic disorders, MRI, quantile regression index, severe mental illness, UK Biobank

## Abstract

Severe mental illnesses (SMI) including major depressive disorder (MDD), bipolar disorder (BD), and schizophrenia spectrum disorder (SSD) elevate accelerated brain aging risks. Cardio‐metabolic disorders (CMD) are common comorbidities in SMI and negatively impact brain health. We validated a linear quantile regression index (QRI) approach against the machine learning “BrainAge” index in an independent SSD cohort (*N* = 206). We tested the direct and additive effects of SMI and CMD effects on accelerated brain aging in the *N* = 1,618 (604 M/1,014 F, average age = 63.53 ± 7.38) subjects with SMI and *N* = 11,849 (5,719 M/6,130 F; 64.42 ± 7.38) controls from the UK Biobank. Subjects were subdivided based on diagnostic status: SMI+/CMD+ (*N* = 665), SMI+/CMD− (*N* = 964), SMI−/CMD+ (*N* = 3,765), SMI−/CMD− (*N* = 8,083). SMI (*F* = 40.47, *p* = 2.06 × 10^−10^) and CMD (*F* = 24.69, *p* = 6.82 × 10^−7^) significantly, independently impacted whole‐brain QRI in SMI+. SSD had the largest effect (Cohen’s *d* = 1.42) then BD (*d* = 0.55), and MDD (*d* = 0.15). Hypertension had a significant effect on SMI+ (*d* = 0.19) and SMI− (*d* = 0.14). SMI effects were direct, independent of MD, and remained significant after correcting for effects of antipsychotic medications. Whole‐brain QRI was significantly (*p* < 10^−16^) associated with the volume of white matter hyperintensities (WMH). However, WMH did not show significant association with SMI and was driven by CMD, chiefly hypertension (*p* < 10^−16^). We used a simple and robust index, QRI, the demonstrate additive effect of SMI and CMD on accelerated brain aging. We showed a greater effect of psychiatric illnesses on QRI compared to cardio‐metabolic illness. Our findings suggest that subjects with SMI should be among the targets for interventions to protect against age‐related cognitive decline.

## Graphical abstract

Severe mental illnesses including major depressive disorder, bipolar disorder, and schizophrenia spectrum disorder elevate accelerated brain aging risks; cardio‐metabolic disorders are common comorbidities and can negatively impact brain health. We used a simple and robust index, QRI, the demonstrate additive effect of SMI and CMD on accelerated brain aging. We showed a greater effect of psychiatric illnesses on QRI compared to cardio‐metabolic illness.

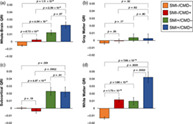

## 1 INTRODUCTION

Patients afflicted with severe mental illness (SMI)—including major depressive disorder (MDD; Kaufmann et al., 2019; Van Camp, van den Ameele, Sabbe, & Oldenburg, 2018), bipolar disorder (BD; Kaufmann et al., 2019; Wolkowitz, Reus, & Mellon, 2011), and schizophrenia spectrum disorder (SSD) (Cetin‐Karayumak et al., 2019; Kaufmann et al., 2019; Kirkpatrick, Messias, Harvey, Fernandez‐Egea, & Bowie, 2008; Kochunov et al., 2016) may experience unfavorable, accelerated aging that elevates the risk for dementia. Previous findings in SSD have reported faster aging‐related decline in the white matter (Cetin‐Karayumak et al., 2019; Kaufmann et al., 2019; Kelly et al., 2018; Kochunov, Ganjgahi, et al., 2016), gray matter subcortical volume (van Erp et al., 2016), and cortical thickness (van Erp et al., 2018) compared to controls. Patients with BD are also at risk for accelerated age‐related decline in white matter (Kaufmann et al., 2019) and the gray matter volumes of the cerebellum (Hallahan et al., 2011), hippocampus (Cao et al., 2016), and prefrontal cortex (Almeida et al., 2009). Furthermore, patients with SSD (Kochunov et al., 2016; Kochunov et al., 2017; Ribe, Laursen, & Charles, 2017) and BD (Gildengers et al., 2007) typically exhibit cognitive deficits, particularly in processing speed and working memory—two domains most commonly affected in dementia. Consequently, this may explain why some SSD (Kochunov et al., 2017; Kochunov, Rowland, et al., 2016; Ribe et al., 2017) and BD (Gildengers et al., 2007) patients are at higher risk for dementia, and have a shorter lifespan even after accounting for premature death due to suicide (Brown, 1997; Tsuang & Woolson, 1978). Findings of accelerated brain aging in MDD have suggested more subtle effects than in SSD and BD (Han et al., 2020; Kaufmann et al., 2019; Schmaal et al., 2017; Wolkowitz et al., 2011). Here, we used a large and representative sample collected by the UK Biobank to evaluate the effects of SMI on individual brain aging trends and to test the hypothesis that accelerated aging in SMI is caused by a combination of SMI and CMD that may be exacerbated by psychiatric medication and alcohol, and/or tobacco use.

There is evidence for a direct link between SMI and accelerated brain aging. Our group, and others, have argued that genetic and environmental risk factors for SMI alter the lifetime cerebral trajectory by causing (a) an earlier than normal age‐of‐peak in brain integrity, (b) lower integrity at the age of peak, and/or (c) an accelerated rate of decline past the age of peak (Kaufmann et al., 2019; Kochunov et al., 2012; Kochunov & Hong, 2014). These risk factors specifically interfere with the developmental trajectories of the frontal, parietal, and temporal areas that continue to develop into the third and fourth decades of life (Kochunov et al., 2011; Kochunov, Ganjgahi, et al., 2016), leading to an earlier onset of cognitive deficits and an elevated (2‐ to 5‐fold) risk for dementia (Ribe et al., 2017). Importantly, accelerated brain aging is not solely due to medication side effects (Han et al., 2020; Van Gestel et al., 2019), and the rate of decline varies across brain tissue types (Han et al., 2020; Wright et al., 2014). Thereby, we hypothesized that accelerated brain aging in SMI may have a cumulative effect, such that SMI and CMD each independently and additively contribute to the accelerated brain aging.

The causes of accelerated brain aging in SMI are unclear, but age‐related cardio‐metabolic disorders (CMD), smoking, alcohol, and socio‐economic factors have been proposed as potential drivers (Brown, 1997; Hennekens, Hennekens, Hollar, & Casey, 2005; Kirkpatrick et al., 2008; Tsuang & Woolson, 1978). Common CMD such as hypertension, diabetes, and high cholesterol, are universal risk factors for unfavorable brain aging and are associated with higher risks of dementia and mortality (Alfaro et al., 2016; Brown, 1997; Hennekens et al., 2005; Kirkpatrick et al., 2008; Tsuang & Woolson, 1978). Aging studies have long associated CMD with the widening of sulci, reduced cortical thickness, enlargement of the lateral ventricles, increased white matter hyperintensity (WMH) burden, and lower hippocampal volumes (Kochunov et al., 2005). Chronic hypertension affects cerebral integrity (Alfaro et al., 2016; Kochunov et al., 2010, 2011, 2012), and is genetically linked with white matter atrophy (Kochunov, Glahn, Lancaster, Winkler, et al., 2010, 2011); it is also associated with the formation of hyperintensive white matter lesions (Kochunov et al., 2009; Kochunov, Glahn, Lancaster, Winkler, et al., 2010). High cholesterol levels are associated with reduced cerebral integrity and density of cerebral arterioles, and lower gray and white matter volumes (Haltia et al., 2007; Walther, Birdsill, Glisky, & Ryan, 2010). Diabetes is likewise a significant risk factor for accelerated brain aging (Biessels, van der Heide, Kamal, Bleys, & Gispen, 2002) and cognitive impairment (Monette, Baird, & Jackson, 2014). Higher prevalence of CMD in SMI may be caused by dietary factors (Dipasquale et al., 2013) and sedentary lifestyle (Daumit et al., 2005). Finally, higher rates of smoking and alcohol consumption in patients with SMI may contribute to accelerated brain aging via direct and cerebrovascular mechanisms (Gons et al., 2011; Kochunov et al., 2013; Zhang, Stein, & Hong, 2011).

Previous studies in accelerated aging in psychiatric illnesses compared patient‐control differences on the aging slopes and evaluated the significance of the diagnosis‐by‐age interaction (Kochunov, Ganjgahi, et al., 2016; Kochunov, Glahn, Rowland, et al., 2012). However, these approaches are focused on group differences and do not provide an index of individual aging assessments. Alternatively, Brain Age approaches predict individual ages from neuroimaging data using machine‐learning approaches trained to draw an association between regional brain measures and chronological age. The Brain Age methods have several shortcomings, including the computational complexity and the lack of stability and translatability of findings across multiple datasets (Smith, Vidaurre, Alfaro‐Almagro, Nichols, & Miller, 2019). We used a new metric, the Quantile Regression Index (QRI), to quantify the impact of SMI on brain aging (J. Lv et al., 2020). QRI is a simple, yet robust modeling approach that can be used to study accelerated aging across multiple diagnostic categories by determining an individual’s position within the expected aging trajectory. Unlike Brain Age, the QRI modeling is linear and requires no training dataset (J. Lv et al., 2020). A positive QRI measure corresponds to accelerated aging, while a negative QRI suggests a delayed aging process (Section 2). We first validated QRI using an established, machine‐learning index called Brain Age, in an independent sample of SSD patients and controls (Jingtao Wang et al., 2020). Then, we used a sample provided by the UK Biobank, to measure direct, additive, and interactive effects on cerebral aging as indexed by QRI. We hypothesized that the direct effects of SMI and CMD on the whole‐brain will correspond to a significant increase in QRI, and that the additive effects of SMI and CMD will show the most severe elevation in subjects with combined SMI and CMD diagnoses. Furthermore, we investigated gray matter, subcortical, and white matter tissue specificity and whether the additive effects are restricted to SSD, BD, and/or MDD.

## 2 METHODS

### 2.1 UK Biobank sample

Neuroimaging (Miller et al., 2016) and clinical data were available for a subset of *N* = 13,467 participants (6,323 M/7,144 F, average age = 64.31 ± 7.38 years). Data were collected between 2006 and 2010 and participants were recruited from the United Kingdom, as part of a large‐scale epidemiological study. All participants provided written informed consent. The full demographic information is available in Table 1. Full medication details are available in Table S1.

**TABLE 1:** Demographic information for the UKBB sample analyzed in this study

Demographic	SMI+			SMI−
	BD	MDD	SSD	
Total number of subjects (M/F)	47 (19/28)	1,590 (591/999)	5 (3/2)	11,849 (5,719/6,130)
Average age ± *SD* (years)	62.93 ± 6.37	63.55 ± 7.40	63.20 ± 9.15	64.42 ± 7.38
*Cardio‐metabolic disorders*				
% Hypertensive subjects (Total/M/F)	30.4% (492/235/257)			24.0% (2,841/1,639/1,202)
% Diabetic subjects (Total/M/F)	5.8% (94/55/39)			0.7% (78/56/22)
% Hyperlipidemic subjects (Total/M/F)	20.1% (325/172/153)			15.1% (1,790/1,116/674)

*Note*: SMI patients were defined as those participants who self‐reported a diagnosis of bipolar disorder (BD), major depressive disorder (MDD), or schizophrenia spectrum disorder (SSD), but were free from any other neuropsychiatric illnesses. Non‐SMI controls reported no psychiatric or neurological illnesses.

#### 2.1.1 Defining patients and controls in the UKBB

We used the UKBB parser software (https://github.com/USC-IGC/ukbb_parser) to identify 1,619 (604 M/1,014 F, age = 63.53 ± 7.38 years) participants with ICD codes corresponding to self‐reported SSD (*N* = 5; 3 M/2 F, age = 63.20 ± 9.15 years), MDD (*N* = 1,590; 591 M/999 F, age = 63.55 ± 7.40 years), and BD (*N* = 47; 19 M/28 F, age = 62.93 ± 6.37 years) diagnoses that were free from neurological disorders and any other psychiatric illnesses. We identified 11,849 subjects (5,719 M/6,130 F, age = 64.42 ± 7.38) as non‐SMI controls, who were free from any self‐reported psychiatric illnesses and neurological conditions.

Similarly, we used the UKBB parser software to identify subjects with ICD codes corresponding to three major CMD: hypertension, diabetes, and hyperlipidemia (Table 1). Of the subjects with SMI, *N* = 492 reported a diagnosis of hypertension, *N* = 94 with diabetes, and *N* = 325 with hyperlipidemia. Non‐SMI controls had *N* = 2,841 with hypertension, *N* = 78 with diabetes, and *N* = 1,790 with hyperlipidemia.

Self‐reported life‐time tobacco smoking (*N* = 361/249 for SMI+/SMI−) and alcohol use were also extracted (*N* = 1,541/11,428 for SMI+/SMI−). All *N* = 5 subjects with SSD were medicated; four were taking antipsychotic medications; one subject also took an antidepressant, and another subject took a mood stabilizer (Table S1). Of the 1,590 MDD patients, 26 were on antipsychotics, 4 on lithium, 495 on antidepressants, and 28 on mood stabilizers. For 47 BD patients, 18 were on antidepressants, 10 were on mood stabilizers, 15 were on lithium, and 14 were on antipsychotics.

#### 2.1.2 Defining subgroups for omnibus testing

For omnibus testing purposes, we subdivided the UKBB sample into four groups based on CMD and SMI diagnostic status: (a) subjects with SMI but free of CMD (SMI+/CMD−; *N* = 964); (b) subjects with SMI and CMD (SMI+/CMD+; *N* = 655); (c) subjects without SMI but with CMD (SMI−/CMD+; *N* = 3,765); and (d) subjects without SMI and CMD (SMI−/CMD−; *N* = 8,083).

### 2.2 Imaging protocol and processing

This study analyzed cortical regional gray matter thickness, gray matter volume in the subcortical structures, and tract‐wise measurements of FA values of the white matter provided by the UKBB. These phenotypes were extracted from neuroimaging data collected with a Siemens Skyra 3 T scanner using a standard 32‐channel RF head coil. The imaging protocol collected high‐resolution T1‐weighted (resolution = 1 × 1 × 1 mm, FOV = 208 × 256 × 256, duration = 5 min, 3D MPRAGE, sagittal, in‐plane acceleration iPAT = 2, prescan‐normalize) and T2 FLAIR images of the brain (resolution = 1.05 × 1 × 1 mm, FOV = 192 × 256 × 256, duration = 6 min, 3D SPACE, sagittal, in‐plane GRAPPA partial phase imaging acceleration factor 2 and partial 7/8 Fourier sampling). Diffusion data were collected with a resolution = 2 × 2 × 2 mm and two diffusion shells of *b* = 1,000 and 2,000 s/mm^2^ with 50 diffusion‐weighted gradient directions per shell, 5 *b* = 0 images, FOV = 104 × 104 × 72, and a duration of 7 min. Imaging data were processed using the UKBB workflow that is based on ENIGMA structural and DTI pipelines. Details of the image preprocessing and analysis are provided by UKBB (Alfaro‐Almagro et al., 2018) and available online at biobank.ctsu.ox.ac.uk/crystal/crystal/docs/brain_mri.pdf. Briefly, 35 measurements of regional cortical GM thickness, 7 measurements of regional gray matter volume, and 24 regional white matter tract FA values per hemisphere were analyzed (Table S2).

### 2.3 Total volume of WMH

Hyperintensive white matter regions observed on T2 weighted FLAIR images reflect accumulation of interstitial fluid and are associated with areas of localized demyelination and white matter damage. We used the WMH data from the UKBB (Field ID 25781), which were automatically estimated with T1 and T2 FLAIR data using the Brain Intensity Abnormality Classification Algorithm in FSL (Griffanti et al., 2016).

### 2.4 Validation sample, imaging, and machine‐learning

A validation sample included 206 healthy controls (137 M/69F, average age = 37.63 years) free of Axis I psychiatric disorders that were age and sex frequency‐matched with *N* = 214 patients with schizophrenia (145 M/69 F, average age = 37.11 years). Full sample demographic information is available elsewhere (Jingtao Wang et al., 2020). All participants gave written informed consent, and the study was approved by the University of Maryland, Baltimore Institutional Review Board. The study focused on DTI data using the average FA values for 26 major white matter tracts extracted by the ENIGMA DTI analysis pipeline (https://www.nitrc.org/projects/enigma_dti; Jahanshad et al., 2013). A BrainAge model was trained using healthy controls and using random forest regression (Breiman, 2001), gradient boosting regression (Friedman, 2001), and LASSO (Least Absolute Shrinkage and Selection Operator; Friedman, Hastie, & Tibshirani, 2010). The BrainAge index was calculated for each participant using the regional DTI data. A measure of Δage (delta age)—sometimes called the “brain age gap”—was calculated as the difference between BrainAge and true chronological age and can be used as a proxy measure of the individual aging process. The full methods for computing the BrainAge are detailed elsewhere (Jingtao Wang et al., 2020). The individual BrainAge estimates of this sample were then used to validate our novel QRI index.

### 2.5 Statistics

All statistical analyses were performed in RStudio (R Core Team, 2020a, 2020b) version 1.2.5033.

#### 2.5.1 Quantile regression analysis—Calculation

**FIGURE 1:**
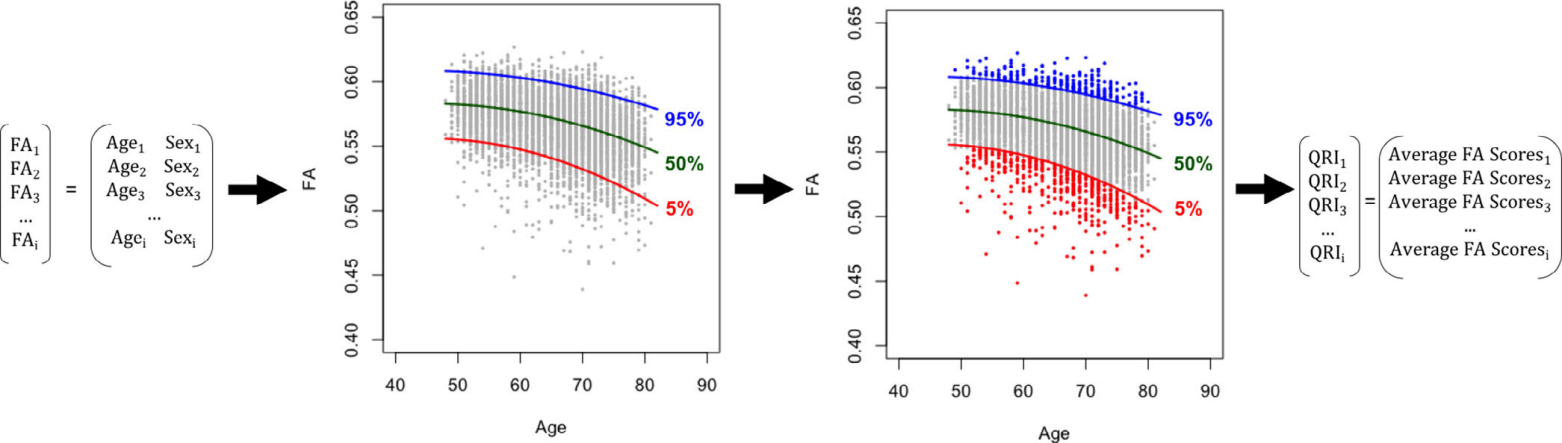
Calculation of the Quantile Regression Index. Quantile regression analysis was performed using the regional data for gray matter thickness, gray matter subcortical volumes, and white matter fractional anisotropy separately. Using FA as an example, the regression analysis generated aging trajectory curves at 5, 50, and 95% for each region. For each subject and each region, the FA value was compared to the 5 and 95% curves to determine placement on the aging trajectory. Values >95% were classified as resistant to aging and assigned a value of −1 (blue); values <5% were considered indicative of accelerated aging and assigned a value of 1 (red); all others were assigned a value of 0. For FA, this resulted in a vector of 22 scores for each subject. The scores were then averaged across the regions to produce an individual’s Quantile Regression Index (QRI) score. The whole‐brain QRI was derived by averaging an individual’s three tissue‐specific QRI scores

Individual assessment of brain aging was performed using quantile regression analysis and deriving a quantile regression index (QRI) score for the regional brain measures (Lv et al., 2020). This functionality is provided in the “QRIpkg” for R (Kochunov, Gao, & Ryan, 2020) available for download at https://CRAN.R-project.org/package=QRIpkg. Briefly, the normative modeling technique provides a map of *individual* deviations from the expected aging trends. The QRI function uses a quantile regression analysis with age serving as a predictor for cortical regional gray matter thickness, subcortical gray matter structure volumes, and white matter fractional anisotropy values to fit three separate models for the 5th, 50th, and 95th percentiles (Figure 1). Then, values for each individual subject are compared to the expected aging trajectory and each regional measure is assigned a score: values >95% of the expected age data were assigned a value of “−1”, indicating an individual’s actual brain age is significantly younger than what is expected for that age; values <5% received a value of “1”, indicating an individual’s actual brain age is significantly older than what is expected for that age; all others were assigned “0”. Regional scores were averaged for cortical thickness, subcortical volume, and white matter to create tissue‐specific QRIs. A whole‐brain QRI was derived by averaging the three tissue‐specific QRIs.

By design, QRI is uncorrelated with age. Additionally, we did not observe a correlation between QRI and intracranial brain volume for any measures but used this value as a covariate when calculating the QRIs. For the validation sample, only QRI for white matter was calculated.

#### 2.5.2 QRI validation

To confirm QRI as a valid measure of accelerated aging, relative to the established BrainAge measure, we performed a linear regression analysis between QRI for white matter with the machine‐learning‐based Δage from a previously published study (Jingtao Wang et al., 2020). We compared QRI and Δage (calculated as the difference between chronological and BrainAge) in the full *N* = 420 validation sample and then in patients (*N* = 214) and controls (*N* = 206) separately. We also calculated the patient‐control effect size for QRI white matter measures, to compare them with the white matter effect size observed in the UKBB sample.

#### 2.5.3 Omnibus testing of significance of effects of psychiatric and CMD

We performed an omnibus test of our hypothesis that SMI without CMD and the CMD without SMI and any other psychiatric illnesses are each independently associated with significantly higher QRI using a two‐way analysis of variance (ANOVA) on the whole‐brain QRI (Equation (1)). We coded predictors: 0 (no diagnosis/substance use) or 1 (diagnosis/substance us) for SMI and cardio‐metabolic disorder (CMD) and tobacco smoking and alcohol. The focus of these analyses was to evaluate the direct versus interactive effects of SMI and CMD on brain aging.

(1) QRI~SMI+CMD+Alcohol+Tobacco+SMI*CMD+SMI*Alcohol+SMI*Tobacco

To further assess the primary hypothesis that SMI and CMD show an additive effect, such that SMI + CMD would have the highest QRI, we performed a *t*‐test to assess the statistical significance of group differences in whole‐brain QRI, for each of the following five scenarios: (I) SMI+/MD− versus SMI−/MD−; (II) SMI−/CMD+ > SMI−/CMD−; (III) SMI+/CMD+ > SMI+/CMD−; (IV) SMI + C/MD+ > SMI−/CMD+; and (V) SMI + C/MD+ > SMI−/CMD−. Additive effects would be fully supported when the five comparisons were all statistically significant in the hypothesized direction. Additive effects may still be present if only some of the above comparisons were significant, but all analyses were in the expected direction.

#### 2.5.4 Testing of medication effects

We used an ANOVA and pairwise Tukey Honest Significant Differences tests to assess the direct and interactive effects of medications (antipsychotic, antidepressant, mood stabilizing, and lithium; coded as 0 or 1; Equation (2)) and CMD in SMI+ subjects.

(2) QRI~Medication+Cardio−metabolic+Medication×Cardio−metabolic

Bonferroni correction to account for Type I errors associated with multiple *N* = 4 QRI comparisons set the significance threshold to *p* < .05/4 = .0125 and trend‐level significance values at .0125 < *p* < .05.

Effect sizes were first calculated for the four QRIs using the *N* = 13,467 sample based on diagnostic status for the three SMI together. The effects of diabetes, hypertension, and hyperlipidemia were analyzed separately in SMI+ and SMI− subjects. Effect sizes were calculated using R package “effsize” (Torchiano, 2020).

### 2.6 Association with WMH

Finally, we tested the association of QRI, SMI, and CMD with the total volume of WMH. We performed univariate correlation analyses among whole‐brain and tissue‐specific QRI and WMH. We also used Equation (1) to test the impact of SMI and CMD on the WMH by using WMH instead of QRI.

## 3 RESULTS

### 3.1 Comparison of QRI and BrainAge Δage in Schizophrenia

**FIGURE 2:**
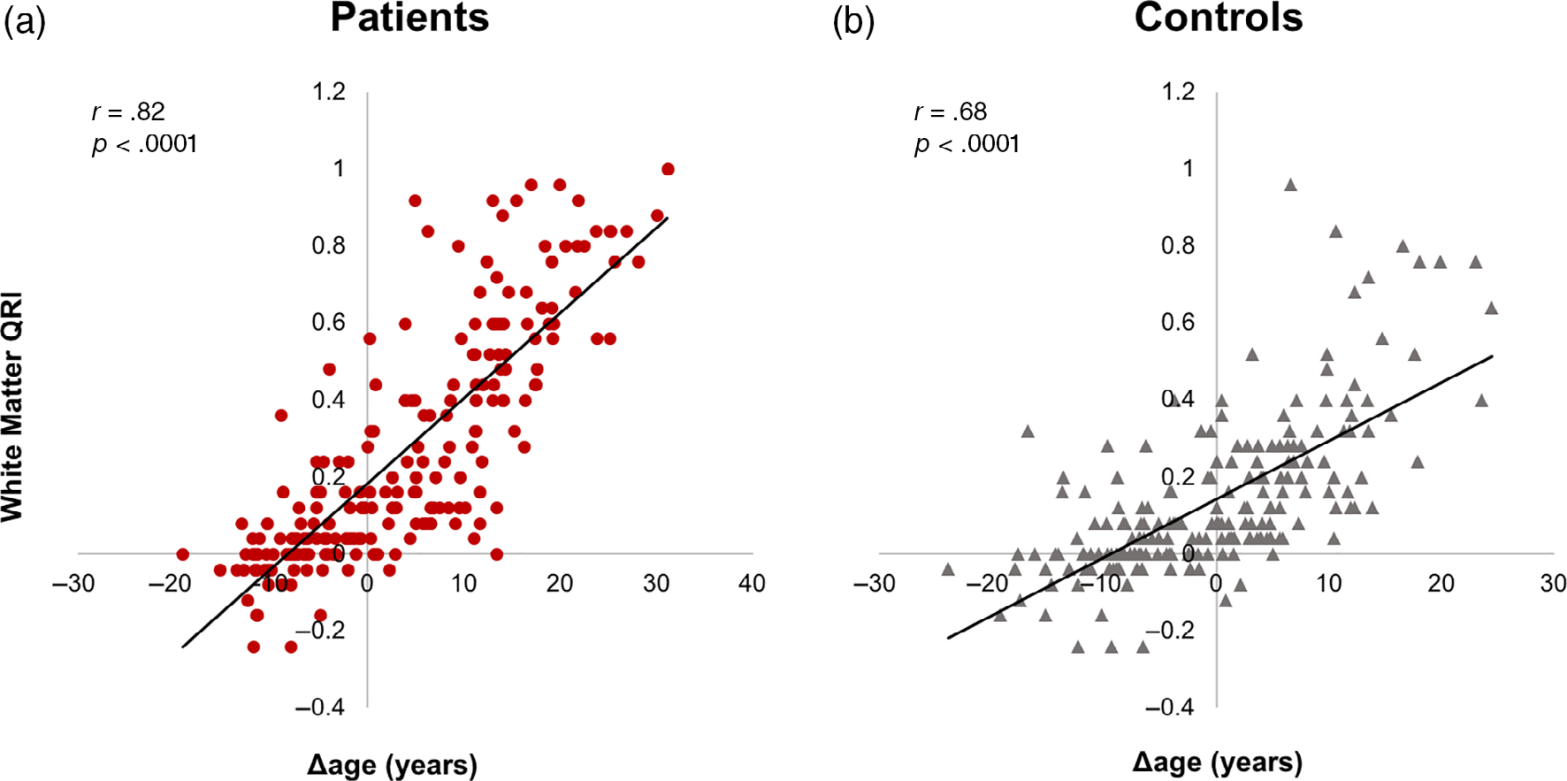
A significant, positive correlation between QRI for White Matter and Δage suggests that a more positive QRI is a sign of more severely accelerated aging. Using the sample of *N* = 206 healthy controls and *N* = 214 patients with SSD from (Jingtao Wang et al., 2020), QRI calculated for white matter FA was significantly and positively correlated with Δage in (a) patients with SSD and (b) controls

We compared QRI to the Δage data calculated using nonlinear, machine learning‐based BrainAge approach published in Jingtao Wang et al. (2020). Neither QRI nor Δage were significantly correlated with chronological age (*r* < .01). Both QRI and Δage demonstrated similar patient‐control effect sizes (Cohen’s *d* = 0.51, 95% CI: [0.32, 0.70]; *d* = 0.41, 95% CI: [0.21, 0.60], for QRI and Δage, respectively) for cerebral white matter in an SSD sample. QRI and Δage were significantly correlated in both patients and controls, separately (Figure 2, *r* = .82 and 0.68, *p* < .0001, respectively).

### 3.2 Accelerated brain aging: SMI and CMD

A two‐way ANOVA analysis showed significant effects of both SMI and CMD on whole‐brain QRI (*F* = 40.47 *p* = 2.06 × 10^−10^; *F* = 24.69, *p* = 6.82 × 10^−7^, for SMI and CMD, respectively) with no significant interactions between them (*p* = .55; Table 2). SMI exerted significant effects on white matter (*F* = 32.51, *p* = 1.21 × 10^−8^) and subcortical QRI (*F* = 28.39, *p* = 1.01 × 10^−7^), but not cortical QRI (*F =* 0.29, *p* = .59; Table 2). CMD demonstrated significant effects on white matter QRI (*F* = 62.80, *p* = 2.60 × 10^−15^). There were no significant interactions between SMI and CMD for any of the imaging modalities (all *p* > .55; Table 2). Alcohol and tobacco smoking status had no direct or interactive effects (all *p* > .07) on the whole brain (Table 2).

**TABLE 2:** SMI and CMD independently contribute to elevated QRI

	QRI gray matter thickness	QRI subcortical volume	QRI White matter	QRI whole brain
SMI	0.29 (0.59)	**28.39 (1.01 × 10** ^**−7**^ **)**	**32.51 (1.21 × 10** ^**−8**^ **)**	**40.47 (2.06 × 10** ^**−10**^ **)**
CMD	0.18 (0.67)	3.37 (0.07)	**62.80 (2.60 × 10** ^**−15**^ **)**	**24.69 (6.82 × 10** ^**−7**^ **)**
Alcohol	0.96 (0.33)	1.79 (0.18)	0.01 (0.91)	0.09 (0.68)
Tobacco	0.10 (0.75)	3.36 (0.07)	0.002 (0.97)	0.86 (0.77)
SMI * CMD	0.05 (0.83)	2.97 (0.09)	0.44 (0.51)	1.35 (0.25)
SMI * Alcohol	1.30 (0.25)	1.10 (0.29)	0.10 (0.75)	1.17 (0.28)
SMI * Tobacco	0.27 (0.60)	0.04 (0.85)	0.19 (0.66)	0.36 (0.55)

*Note*: *F* (*p*‐value). Bolded values indicate significance (*p* < .05/4 = .0125).

**FIGURE 3:**
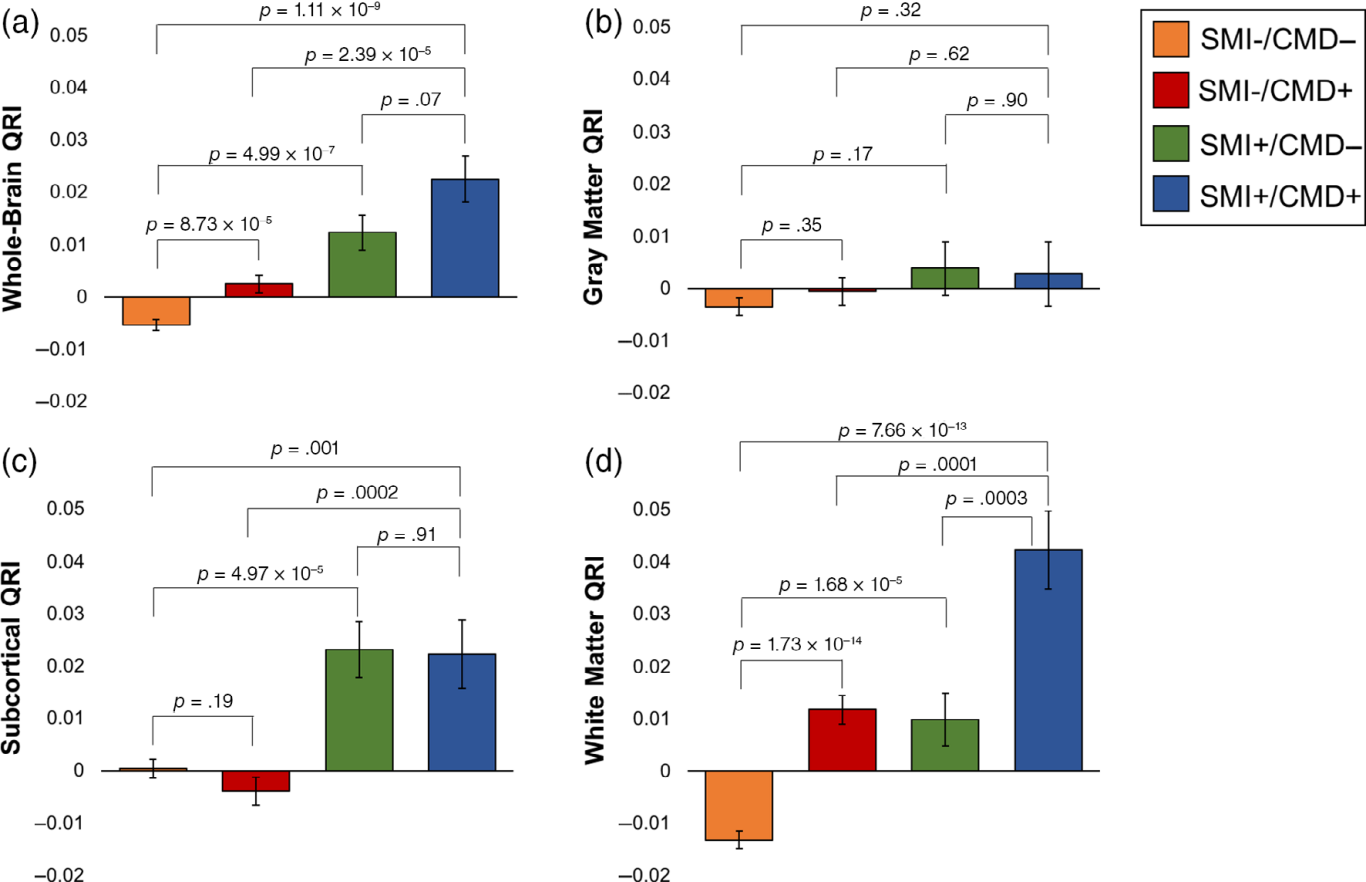
The additive effects of SMI and CMD on accelerated aging on the (a) whole‐brain QRI, (b) gray matter thickness QRI, (c) subcortical volume QRI, and (d) white matter QRI. A *t*‐test to assess statistical significance was performed for the following five scenarios: (I) SMI+/CMD− versus SMI−/CMD−; (II) SMI−/CMD+ > SMI−/CMD−; (III) SMI+/CMD+ > SMI+/CMD−; (IV) SMI+/CMD+ > SMI−/CMD+; and (V) SMI+/CMD+ > SMI−/CMD−

Group comparisons to SMI−/CMD− showed a significant stepwise increase in average whole‐brain QRI (Figure 3a). SMI+/CMD+ had the largest average QRI (*t* = 6.34, *p* = 3.90 × 10^−10^; Figure 3a) followed by SMI+/CMD− (*t* = 4.10; *p* = 4.25 × 10^−5^) and SMI−/CMD+ (*t* = 4.15; *p* = 3.30 × 10^−5^). SMI−/CMD− had the lowest average QRI.

Tissue‐specific QRI comparisons are shown in Figure 3b–d. Cortical gray matter thickness QRI showed no significant group differences or additive effects (Figure 3b, all *p* > .66). Subcortical gray matter volume QRIs showed significant group differences for SMI−/CMD− versus SMI+/CMD+ (*p* = 2.94 × 10^−7^), SMI+/CMD+ versus SMI−/CMD+ (*p* = 8.51 × 10^−6^), and SMI+/CMD− versus SMI−/CMD− (*p* = .002) but not SMI−/CMD− versus SMI−/CMD+ (*p* = .28) suggesting that QRI elevation is associated with SMI but not CMD. The lack of a detectable difference in QRI between SMI+/CMD− and SMI+/CMD+ (*p* = .02) further supported this finding (Figure 3c). White matter QRI showed significant group differences in all five group comparisons, suggesting that SMI and CMD are independently and additively associated with accelerated aging (all *p* < .001, Figure 3d).

### 3.3 Disorder‐specific effects: SMI

**FIGURE 4:**
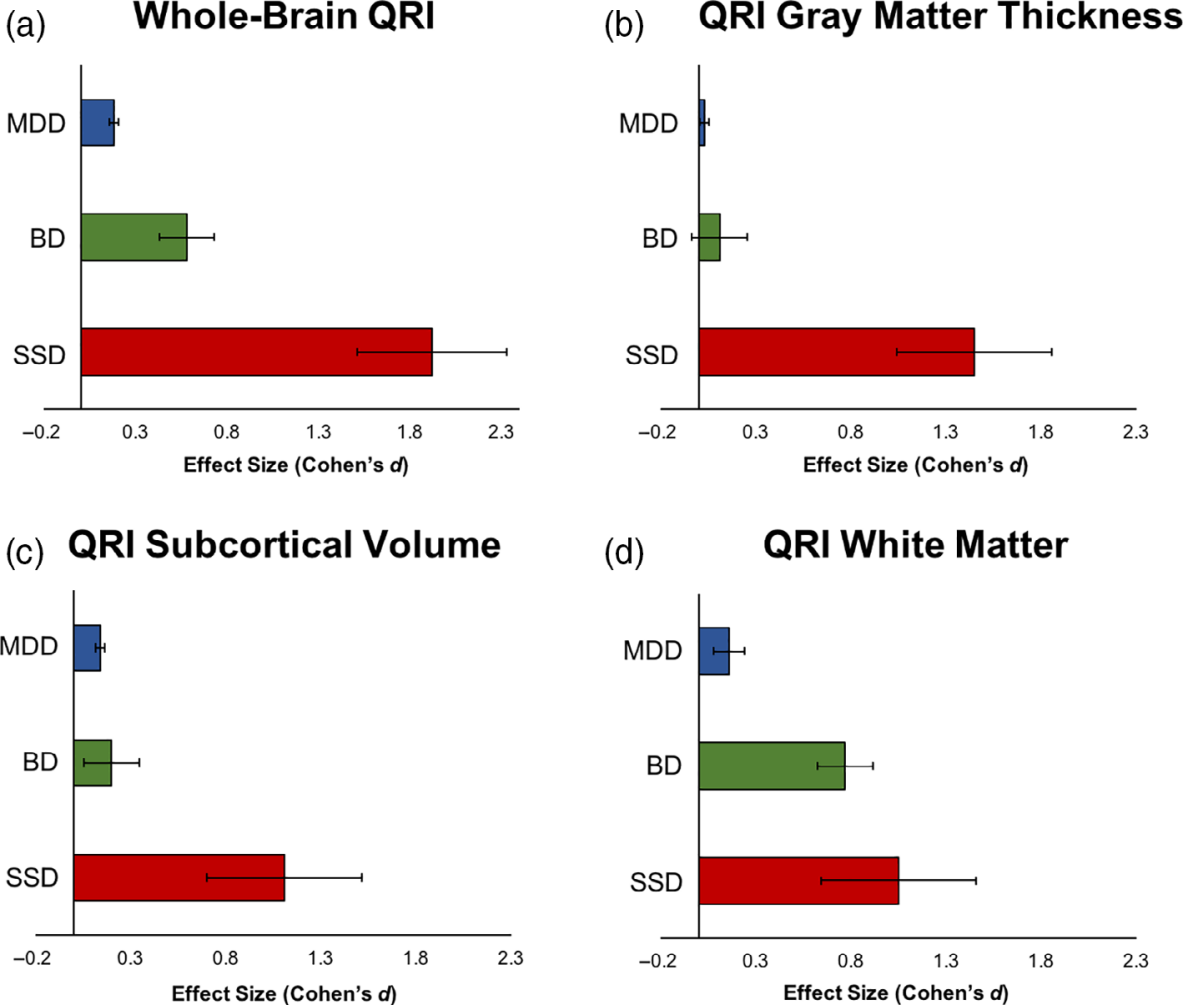
SMI+ patients show significantly increased QRI compared to SMI− subjects. Effect sizes for (a) whole‐brain QRI, (b) gray matter thickness, (c) subcortical volume, and (d) white matter

Given the lack of a detectable interaction between SMI and CMD, these effects were tested separately in the full sample. Effect sizes of whole‐brain QRI for each of the three SMI diagnoses are shown in Figure 4 and Table S3. Positive effect sizes indicated that SMI were associated with older looking brains. SSD had the largest effect size for whole‐brain QRI (Figure 4a, Cohen’s *d* = 1.42; 95% CI: [0.54, 2.30]) followed by BD (*d* = 0.55; 95% CI: [0.26, 0.84]) and MDD (*d* = 0.15; 95% CI: [0.10, 0.21]).

For cortical thickness QRI (Figure 4b), the effects of MDD, SSD, and BD were not significant.

For subcortical gray matter volume QRI, subjects with SSD, BD, and MDD showed significant effect sizes for subcortical volume (Figure 4c, *d* = 1.49, 95% CI: [0.62, 2.38]; *d* = 0.42, 95% CI: [0.13, 0.71]; *d* = 0.13, 95% CI: [0.08, 0.18], respectively).

For white matter QRI, BD (Figure 4d, *d* = 0.69; 95% CI: [0.40, 0.97]) and MDD (*d* = 0.14, 95% CI: [0.09, 0.19]) showed significant effect sizes.

### 3.4 Disorder‐specific effects: MD

**FIGURE 5:**
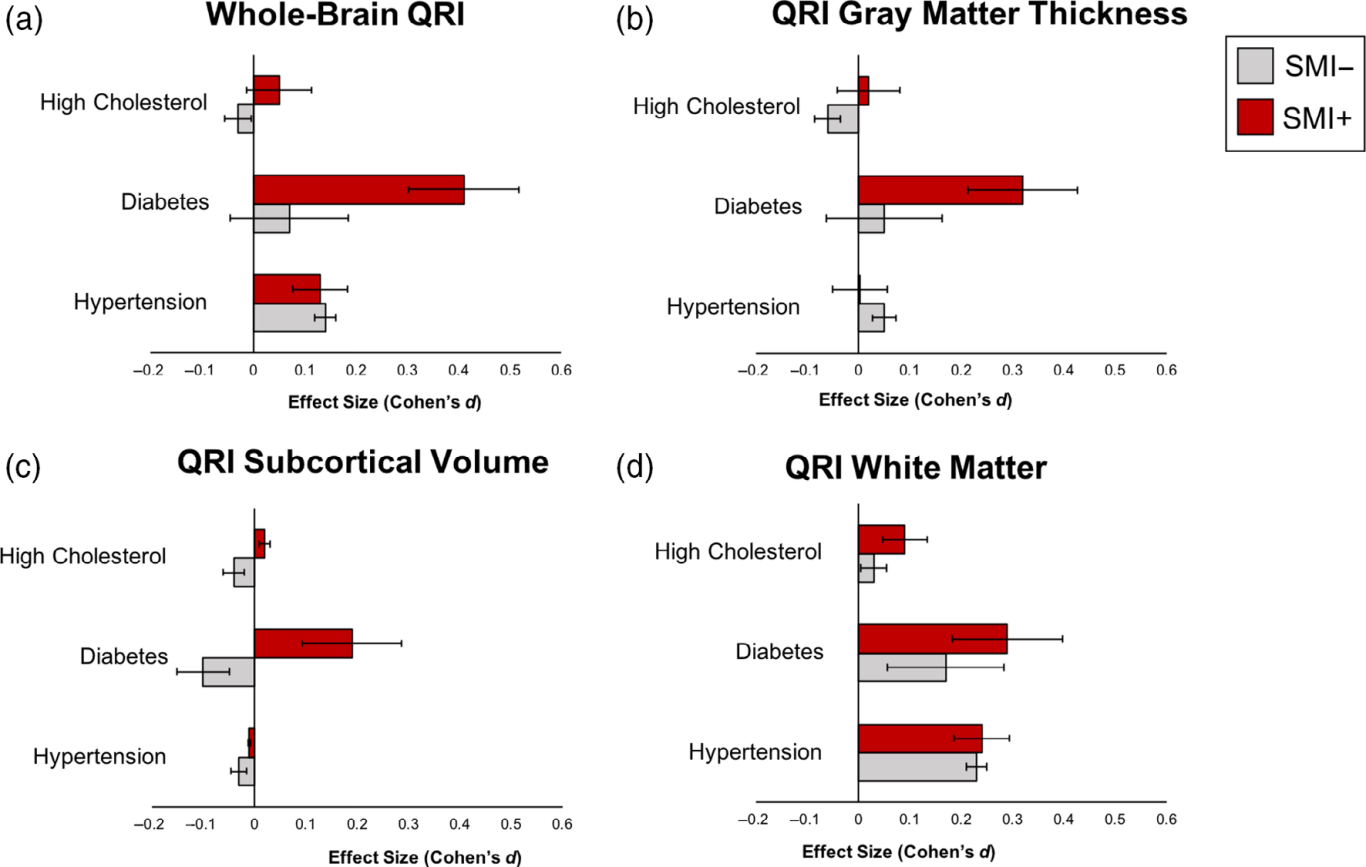
Effects of common cardio‐metabolic disorders on whole‐brain and tissue‐specific QRI in SMI+ (red) and SMI− (gray)

The effect sizes for each of the three cardio‐metabolic conditions on the whole brain QRI were calculated separately in SMI+ (*N* = 1,619) and SMI− (*N* = 11,849; Figure 5a and Table S4). Hypertension showed significant and comparable effects for SMI+ and SMI− subjects (*d* = 0.19, 95% CI: [0.08, 0.29]; *d* = 0.14, 95% CI: [0.09, 0.18], respectively). Diabetes showed a significant effect size for those SMI+ (*d* = 0.41, 95% CI: [0.20, 0.62]), but not for SMI− (*d* = 0.11, 95% CI: [−0.12, 0.33]). High cholesterol showed no significant effects in either group. The effects of CMD on tissue‐specific QRI are shown in Figure 5b–d and Table S4.

### 3.5 Effects of antipsychotic, antidepressant, mood stabilizers, lithium, tobacco, and alcohol

Antipsychotics were the only medications with significant direct effects on subcortical and whole‐brain QRIs (*p* = 2.24 × 10^−3^, 8.28 × 10^−3^, respectively) and suggestive effects on WM QRI (*p* = .02). A significant antidepressant–CMD interactive effect was observed for subcortical QRI (*p* = .01), suggestive of accelerated aging (Table S5).

A post hoc analysis (Table S6) that separated the three CMD demonstrated significant positive interactions between antipsychotic medication and diabetes on the whole‐brain (*p* = .01) and subcortical QRIs (*p* = 2.72 × 10^−3^). Lithium showed a positive interaction with hypertension (*p* = 3.12 × 10^−3^) and a negative interaction with high cholesterol (*p* = 4.73 × 10^−5^) on the WM QRI. Specific group comparisons are presented in Table S7.

### 3.6 Association among QRI, SMI, and CMD and WMH

In the full sample, the whole‐brain QRI was highly correlated with whole‐brain WMH volume (*r* = .18, *p* < 10^−16^). This association was primarily driven by QRI‐WM (*r* = .33, *p* < 10^−16^), however, correlations with QRI‐cortical and subcortical were also significant (*r* = .02 and .03, *p* < .001). Equation (1) was highly significant when WMH was used instead of QRI (*F* = 139.8, *p* < 10^−16^), however, SMI was not a significant predictor (*t* = 1.9, *p* = .06). CMD, smoking, and alcohol use highly associated with higher WMH (*t* = 24.0, 5.8, and 3.1, respectively, *p* < .001). There were no significant interactive effects between SMI and substance on the WMH. CMD‐specific analyses demonstrated that all three contributed to the higher WMH load (*t* = 19.7, 6.9 and 7.4, for hypertension, diabetes, and hyperlipidemia, respectively, *p* < 10^−12^).

## 4 DISCUSSION

We evaluated the effects of three SMI—SSD, MDD, and BD, and three common CMD—on brain aging in a large and representative sample provided by the UK Biobank. We used a novel quantile regression index (QRI) to quantify accelerated or delayed brain aging in an individual and validated it against a more established BrainAge index, Δage. We show that SMI is significantly associated with accelerated brain aging and this effect is additive and independent of CMD. We replicated the effect of CMD on brain aging and observed similar trends in both SMI+ subjects and controls. The effects of SMI were direct and remained after correction for psychiatric medications and substance use. However, there were some significant interactions between antipsychotic medication and cardio‐metabolic illnesses that suggested a possible contribution of antipsychotic medications to cardio‐metabolic dysregulation. Treatment with lithium did suggest a significant neuroprotective effect. In summary, we observed intriguing patterns of accelerated aging that varied by disorder and brain tissues. These effects were direct and suggested that subjects with SMI are vulnerable to unfavorable, accelerated brain aging.

We first validated that QRI captures most of the variance obtained using the traditional but more computationally complex, machine‐learning BrainAge method. Using an independent sample of SSD patients (Jingtao Wang et al., 2020), we showed a strong positive correlation between white matter QRI and Δage in both patients and controls. QRI and Δage showed a similar effect of diagnosis. After validation, we used the UKBB sample to show that mental illness is directly associated with accelerated brain aging and can be used as a biomarker to study these changes. The effects of mental illness were stronger than, additive to, and independent of, cardio‐metabolic illnesses. We observed significant direct effects of antipsychotic medication and cardio‐metabolic illnesses. Specifically, the significant effects of antipsychotic medication in subjects with diabetes suggest that careful consideration should be given to the long‐term side effects of these medications alongside the consideration of immediate side effect risks. In summary, SMI is a significant risk for unfavorable cerebral aging and patients with SMI should be the primary target of preventative interventions against accelerated aging.

Accelerated brain aging in patients with SMI has been suggested since Kraepelin’s definition of psychotic disorders as “premature dementia” and is supported by the significantly higher risk of aging‐related neurocognitive disorders in patients with SMI compared to the general population (Kirkpatrick et al., 2008; Ribe et al., 2017; Wolkowitz et al., 2011). Here, we used a statistically powerful epidemiological sample to study the effects of SMI on aging. We first demonstrated significant SSD aging effects in an independent sample and subsequently for SSD, MDD, and BD in the UKBB sample. While these three disorders may lack the neuropathological findings common in Alzheimer’s disease, neuroimaging studies have identified similarities in deficit patterns between SMI and dementia that mirror the pattern of cognitive deficits (Kochunov et al., 2020; P. Kochunov et al., 2020). For example, anatomical integrity in the temporal lobe, hippocampal regions, and white matter tracts that connect them are highly impacted in many psychiatric illnesses including SSD, BD, and MDD (Crossley et al., 2014; P. Kochunov, Zavaliangos‐Petropulu, et al., 2020; Schmaal et al., 2016; van Erp et al., 2016). The effect size observed in this study replicated the pattern of the severity of accelerated aging in SMI in the analysis performed by Kaufmann et al. (2019). They likewise reported the SSD > BD > MDD patterns of aging with effect sizes (*d* = 0.51, 0.29, and 0.10) that were numerically smaller but not much different from the effect sizes observed by this study. The study by Kaufmann did not find significantly accelerated aging in MDD. This is likely due to a small sample (*N* = 208) of MDD subjects in their analysis. In addition, the study by Kaufmann did not include white matter measurements which showed the largest evidence of accelerated aging in this analysis.

The effect sizes for SMI on aging were numerically larger than those for CMD that impact brain integrity through cerebrovascular and cardio‐metabolic causes—hypertension, hyperlipidemia, and diabetes (Marks, Katz, Styner, & Smith, 2010; Spieker et al., 2015). We observed significant and similar effect sizes for hypertension for the whole‐brain (*d* = 0.19, 0.14) and white matter (*d =* 0.22, 0.21) QRIs in subjects with SMI and controls. We readily replicated hypertension as a specific risk factor for reduced cerebral white matter integrity. Chronic hypertension is associated with damage to the long‐penetrating cerebral blood vessels (Kochunov, Glahn, Lancaster, Winkler, et al., 2010) that leads to reduced white matter integrity, formation of hyperintense lesions (Jagust, Harvey, Mungas, & Haan, 2005), and eventually deficits in cortical thickness and subcortical volumes (Alfaro et al., 2016; Kochunov et al., 2011, 2012; Kochunov, Glahn, Lancaster, Winkler, et al., 2010). The effects of hyperlipidemia were not significant in both groups. Hyperlipidemia has been associated with reduced cerebral integrity. However, these effects become inconsistent once controlled for hypertension and other illnesses (Anstey, Ashby‐Mitchell, & Peters, 2017). For example, some studies have linked hyperlipidemia with reduced performance on cognitive assessments (Meusel et al., 2017) and a higher risk for dementia and Alzheimer’s disease (Solomon et al., 2009). Conversely, others have shown that higher cholesterol levels are associated with better cognitive performance (Lv et al., 2016) and a lower risk for dementia (Reitz et al., 2010); yet still others have reported no relationship (Mielke et al., 2010).

Diabetes is likewise a significant risk factor for accelerated brain aging (Biessels et al., 2002), but in this study, its effects were only detectable in patients with SMI. Detrimental effects of diabetes were observed for gray matter subcortical volume, white matter, and whole‐brain QRI. We tested the hypothesis that subjects with SMI may be more sensitive to diabetes due to antipsychotic medication side effects, specifically high blood sugar (Foley & Morley, 2011; Perez‐Iglesias et al., 2014), that can exacerbate the effects of diabetes on the brain. We observed significant direct effects of antipsychotic medication on the subcortical and whole‐brain QRI and interaction effects of antidepressants and CMD on subcortical QRI. Subjects who took antipsychotic medications and were diagnosed with diabetes were at a higher risk of accelerated aging. However, this outcome may be biased by the higher use of antipsychotic medications in SSD and BD, two disorders with more greatly accelerated aging rates, compared to MDD (only 27 out of 1,590 MDD subjects took antipsychotic medications). Nonetheless, the interaction suggests that careful consideration of the cardio‐metabolic side effects of antipsychotics is necessary in subjects with existing diabetes. We observed no significant direct or interaction effects for lithium or mood stabilizers. Lithium is a common medication prescribed to treat BD. The use of lithium medication has been associated with greater gray matter density, giving rise to the hypothesis that lithium could be used as a positive modulator of aging (Bearden et al., 2007; Monkul et al., 2007; Moore et al., 2009). In summary, the effects of the mental illnesses remained strong and significant and only modestly explained using psychiatric medications.

Finally, we evaluated association of QRI with an independent neuroimaging index of aging—the volume of WMH, as well as impact of SMI and CMD on WMH. The WMH are regions of accumulation of extra‐cellular water due to focal degradation of the myelin sheath (Fazekas et al., 1993) and their volume is an important neuroimaging marker of white integrity (DeStefano et al., 2006; Kochunov et al., 2008, 2009; Kochunov, Glahn, Lancaster, Winkler, et al., 2010; Turner et al., 2005). As expected QRI‐WM showed the strongest association with WMH, with the indexes sharing ~11% of the variance. The CMD were the strongest predictors of the elevated WMH, while SMI was not a significant predictor. We found that hypertension showed the strongest association with WMH, which replicates previously reported genetic links between this illness and the integrity of cerebral white matter (Kochunov et al., 2009, 2010; Kochunov, Glahn, Lancaster, Winkler, et al., 2010). The lack of association between SMI and WMH is likewise in agreement with previously reported findings of modest‐to‐nonsignificant differences in WMH among people with SMI and controls (Beyer, Young, Kuchibhatla, & Krishnan, 2009; Kochunov et al., 2014; Wadhwa et al., 2019; Zanetti et al., 2008).

This study has several methodological and conceptual limitations. QRI was used in this analysis as alternative to the BrainAge/Gap indices. QRI is a simple index that is based on evaluation of the statistical deviation from the populational trends and requires no preliminary training. The BrainAge/Gap indices use complex machine learning approaches to derive the relationship between age and variance in neuroimaging traits. We observed a strong correlation between QRI and Δage in one disease (SSD) and in healthy controls but that does not necessarily prove that these findings can be translated to other illnesses. Second, *the UKBB recruitment is biased toward healthy volunteers which reduces the number of participants with mental illness/substance use versus the population prevalence* (Fry et al., 2017). The sample used included only a small number of subjects with SSD and therefore lacks statistical power in those results. However, the effect sizes in SSD were verified in an independent SSD cohorts and effects of SSD on the whole‐brain QRI were significant. Furthermore, the UKBB sample had many people with MDD and demonstrated significant effects in that illness as well as for BD; the sample sizes for the three CMD varied in size. Third, the *diagnostic information provided in UKBB is based on self‐reported information and hospitalization records and may be susceptible to misclassifications* (Bycroft et al., 2018). Additionally, the CMD diagnoses were based on ICD codes which may not have captured the effects of treated versus untreated CMD. Ad‐hoc analyses that identified subjects with elevated blood pressure (SBP and DBP > 140 and 90 mmHg) demonstrated similar effect sizes but were not included due to much smaller sample for whom two or more blood pressure measurements were available. Our analyses focused on the independent versus interactive effects of SMI and CMD. It is common for CMD to cluster in individuals and further analyses would be needed to study the additive versus interactive effects of CMD on brain aging.

At this time, the full UKBB population is comprised of approximately 54% female and 46% male subjects. As such, our sample mirrored the distribution and contained fewer male subjects likely due to the lack of male imaging data collected and increased MDD diagnosis in females. Post hoc analyses revealed significant sex effects for white matter, subcortical, and whole‐brain QRI that did not significantly change the impact of SMI and CMD on QRI. Furthermore, male subjects showed a significantly larger QRI compared with females which agrees with previous studies performed by the UKBB (Ritchie et al., 2018) and ENIGMA (Wierenga et al., 2020) that reported significant structural differences and increased variability in males compared to females. This UKBB sample is a cross‐sectional study, and as such, cross‐sectional estimates may not follow longitudinal trends (Bycroft et al., 2018). There also exists the potential survival bias within the sample since the age of subjects ranged from 50 to 80 years. Finally, the sample was predominately British or Irish (93%). Expanding the sample to include younger, ethnically diverse subjects as well as longitudinal data would allow for QRI to be tracked over time in a more representative sample and potential interventions tested.

## 5 CONCLUSIONS

In conclusion, we used a simple, yet robust, quantile regression index (QRI) to evaluate accelerated aging using a large sample from the UK Biobank. We successfully validated the QRI approach against the commonly used machine‐learning BrainAge methods in an independent SSD sample. In the UKBB sample, we showed significant omnibus effects of mental illness and CMD with no significant interaction between them. We compared the effect of psychiatric (SSD, MDD, and BD) and cardio‐metabolic (diabetes, hyperlipidemia, and hypertension) disorders on cortical gray matter thickness, subcortical gray matter volume, white matter, and the whole brain. We showed a greater effect of psychiatric illnesses on QRI compared to cardio‐metabolic illness. Progress in medical care has improved the quality of life for many patients with psychiatric illness, but the personal and economic costs of these disorders may be further worsened by the high risk of brain deterioration and faster cognitive decline throughout their lifetime. Our findings suggest that patients with SSD, BD, and MDD should be a primary target for interventions to protect against age‐related cognitive decline. It also calls for interventions to protect against burdens imposed from common cardio‐metabolic conditions, especially hypertension, on the central age‐related decline.

## CONFLICT OF INTEREST

L. Elliot Hong has received or plans to receive research funding or consulting fees on research projects from Mitsubishi, Your Energy Systems LLC, Neuralstem, Taisho, Heptares, Pfizer, Luye Pharma, Sound Pharma, Takeda, and Regeneron. None was involved in the design, analysis, or outcomes of the study. Paul Thompson and Neda Jahanshad received grant support from Biogen, Inc. (Boston, MA) for research unrelated to the topic of this article. All other authors declare no conflicts of interest.

## Supporting information


**Appendix S1**: Supporting Information

## Data Availability

Data will be made available by request through material sharing agreement.
