# Supplementary material for: The additive impact of cardio‐metabolic disorders and psychiatric illnesses on accelerated brain aging
Source: Hum Brain Mapp. 2022 Feb 3;43(6):1997–2010. doi: 10.1002/hbm.25769 (PMC8933252; doi:10.1002/hbm.25769)
Supplement: Supplementary file 1 — Appendix S1: Supporting Information [file HBM-43-1997-s001.docx]

**The Additive Impact of Cardio-Metabolic Disorders and Psychiatric Illnesses**

**on Accelerated Brain Aging**

Meghann C. Ryan MS^1^, L. Elliot Hong MD^1^, Kathryn S. Hatch BS^1^, Si Gao MS^1^, Shuo Chen PhD^1,2^, Krystl Haerian MD^3^, Jingtao Wang MS^1,4^, Eric L. Goldwaser DO, PhD^1^, Xiaoming Du PhD^1^, Bhim M. Adhikari PhD^1^, Heather Bruce MD^1^, Stephanie Hare PhD^1^, Mark D. Kvarta MD, PhD^1^, Neda Jahanshad PhD^5^, Thomas E. Nichols PhD^6^, Paul M. Thompson PhD^5^, Peter Kochunov PhD^1^

1. Maryland Psychiatric Research Center, Department of Psychiatry, University of Maryland School of Medicine, Baltimore, MD, USA
2. Division of Biostatistics and Bioinformatics, Department of Public Health and Epidemiology, University of Maryland School of Medicine, Baltimore, MD, USA
3. Department of Clinical Research and Leadership, School of Medicine and Health Sciences, George Washington University
4. Department of Biostatistics, School of Public Health, Cheeloo College of Medicine, Shandong University
5. Imaging Genetics Center, Stevens Neuroimaging & Informatics Institute, Keck School of Medicine of USC, Marina del Rey, CA, USA
6. Nuffield Department of Population Health of the University of Oxford, Oxford, United Kingdom

**Corresponding Author**

Dr. Peter Kochunov

Maryland Psychiatric Research Center

Department of Psychiatry

University of Maryland, School of Medicine

Baltimore, MD, USA

**Phone:** (410) 402-6110

**E-mail:** [pkochunov@som.umaryland.edu](mailto:pkochunov@som.umaryland.edu)

**Fax:** (410) 402-6778

**Running Title:** Brain Aging in Metabolic and Psychiatric Illness

**SUPPLEMENTAL MATERIAL**

This supplemental file contains **Tables S1-S7**.

**Table S1.** Detailed medication use in patients with severe mental illness. N=44 were taking antipsychotic medications. N=32 reported taking one antipsychotic; N=12 reported two antipsychotics. N=55 patients were using mood stabilizers. N=53 were taking one mood stabilizers while N=2 were taking two. N=309 patients were using antidepressants with N=299 only taking one and N=10 taking two antidepressants

| **Type** | **Medication** | **Number of Subjects** |
| --- | --- | --- |
| Antipsychotic | Chlorpromazine | 2 |
|  | Clozaril | 1 |
|  | Fluanxol | 2 |
|  | Flupentixol | 1 |
|  | Lithium | 16 |
|  | Olanzapine | 12 |
|  | Prochlorperazine | 5 |
|  | Promazine | 2 |
|  | Quetiapine | 11 |
|  | Seroquel | 2 |
|  | Stelazine | 1 |
|  | Trifluoperazine | 2 |
| Antidepressant | Sertraline | 43 |
|  | Prozac | 5 |
|  | Citalopram | 145 |
|  | Escitalopram | 15 |
|  | Paroxetine | 19 |
|  | Duloxetine | 6 |
|  | Venlafaxine | 35 |
|  | Amitriptyline | 4 |
|  | Clomipramine | 9 |
|  | Imipramine | 3 |
|  | Tofranil | 0 |
|  | Trimipramine | 2 |
|  | Nortriptyline | 3 |
|  | Trazodone | 10 |
|  | Mirtazapine | 20 |
| Mood Stabilizer | Sodium Valproate | 43 |
|  | Lamotrigine | 4 |
|  | Carbamazepine | 2 |
|  | Lamictal | 1 |
|  | Gabapentin | 7 |

**Table S2.** ANOVA for patients with SMI and controls. Data was collected using a Siemens Skyra 3T scanner. **Bolded** values indicate significance after Bonferroni correction for multiple comparisons. Gray matter cortical thickness: p<0.05/33=0.0015. White matter: p<0.05/22=0.002. Gray matter subcortical volume: p<0.05/8=0.006. *Italicized* values indicate trend level significance. Gray matter cortical thickness: 0.05>p>0.0015. White matter: 0.05>p>0.002. Gray matter subcortical volume: 0.05>p>0.006.

| ***Gray Matter Cortical Thickness*** | ***F* value (*P* value)** |
| --- | --- |
| Banks of Superior Temporal Sulcus | 1.02 (0.31) |
| Caudal Anterior Cingulate Cortex | 1.34 (0.25) |
| Caudal Middle Frontal Gyrus | 0.02 (0.90) |
| Cuneus | 0.19 (0.67) |
| Entorhinal Cortex | 0.18 (0.67) |
| Fusiform Gyrus | 0.31 (0.58) |
| Inferior Parietal Cortex | 0.70 (0.40) |
| Inferior Temporal Gyrus | 1.29 (0.26) |
| Isthmus Cingulate Cortex | 1.40 (0.24) |
| Lateral Occipital Cortex | 1.59 (0.21) |
| Lateral Orbitofrontal Cortex | 0.18 (0.67) |
| Lingual Gyrus | 0.94 (0.33) |
| Medial Orbitofrontal Cortex | 0.93 (0.33) |
| Middle Temporal Gyrus | *4.82 (0.03)* |
| Parahippocampal Gyrus | 1.76 (0.18) |
| Paracentral Lobule | 0.10 (0.75) |
| Pars Opercularis of Inferior Frontal Gyrus | 1.61 (0.20) |
| Pars Orbitalis of Inferior Frontal Gyrus | 0.26 (0.61) |
| Pars Triangularis of Inferior Frontal Gyrus | 0.04 (0.84) |
| Pericalcarine Cortex | 3.23 (0.07) |
| Postcentral Gyrus | 1.38 (0.24) |
| Posterior Cingulate Cortex | 1.14 (0.29) |
| Precentral Gyrus | 0.35 (0.55) |
| Precuneus | 0.30 (0.58) |
| Rostral Anterior Cingulate Cortex | 0.47 (0.49) |
| Rostral Middle Frontal Gyrus | 0.22 (0.64) |
| Superior Frontal Gyrus | 0.96 (0.33) |
| Superior Parietal Cortex | 3.48 (0.06) |
| Superior Temporal Gyrus | 0.56 (0.46) |
| Supramarginal Gyrus | 0.08 (0.78) |
| Frontal Pole | 0.03 (0.87) |
| Transverse Temporal Gyrus | 3.40 (0.07) |
| Insula | 1.67 (0.20) |
| ***White Matter*** | |
| Internal Capsule (IC) | 0.23 (0.63) |
| Genu of Corpus Callosum (GCC) | **22.84 (1.78⋅10^-6^)** |
| Body of Corpus Callosum (BCC) | **12.88 (3.34⋅10^-4^)** |
| Splenium of Corpus Callosum (SCC) | *6.70 (9.66⋅10^-3^)* |
| Fornix (FX) | 0.63 (0.43) |
| Cortico-Spinal Tract (CST) | 3.38 (0.07) |
| Anterior Limb of Internal Capsule (ALIC) | **19.87 (8.36⋅10^-6^)** |
| Posterior Limb of Internal Capsule (PLIC) | *4.28 (0.04)* |
| Retrolenticular Limb of the Internal Capsule (RLIC) | *4.54 (0.03)* |
| Anterior Corona Radiata (ACR) | *6.18 (0.01)* |
| Superior Corona Radiata (SCR) | **10.10 (1.49⋅10^-3^)** |
| Posterior Corona Radiata (PCR) | *4.85 (0.03)* |
| Posterior Thalamic Radiation(PTR) | **10.95 (9.37⋅10^-4^)** |
| Sagittal Striatum (SS) | *7.96 (4.80⋅10^-3^)* |
| External Capsule (EC) | **9.97 (1.59⋅10^-3^)** |
| Cingulum (CGC) | **12.35 (4.41⋅10^-4^)** |
| Cingulum hippocampus gyrus (CHG) | 1.28 (0.26) |
| Fornix-Stria Terminalis (FXST) | *5.25 (0.02)* |
| Superior Longitudinal Fasciculus (SLF) | *4.10 (0.04)* |
| Superior Fronto-Occipital Fasciculus (SFO) | **11.90 (5.64⋅10^-4^)** |
| Uncinate Fasciculus (UNC) | **14.86 (1.16⋅10^-4^)** |
| Tapetum (TAP) | 0.02 (0.88) |
| ***Subcortical Volume*** | |
| Lateral Ventricle | 1.36 (0.24) |
| Thalamus | **35.21 (3.03⋅10^-9^)** |
| Caudate | **18.71 (1.53⋅10^-5^)** |
| Putamen | **13.48 (2.42⋅10^-4^)** |
| Palladium | **27.62 (1.50⋅10^-7^)** |
| Hippocampus | *4.81 (0.03)* |
| Amygdala | *5.77 (0.02)* |
| Accumbens | **10.05 (1.52⋅10^-3^)** |

**Table S3.** Effect Sizes [Confidence Intervals] for subjects SMI+ vs. those SMI-.

SSD: Schizophrenia Spectrum Disorder

BD: Bipolar Disorder

MDD: Major Depressive Disorder

| **Diagnosis** | **Gray Matter Thickness QRI** | **Subcortical Volume QRI** | **White Matter QRI** | **Whole-brain QRI** |
| --- | --- | --- | --- | --- |
| SSD | 0.54 [-0.34, 1.41] | **1.50 [0.62, 2.38]** | 0.49 [-0.39, 1.36] | **1.42 [0.54, 2.30]** |
| BD | -0.09 [-0.37, 0.20] | **0.43 [0.13, 0.71]** | **0.69 [0.40, 0.97]** | **0.55 [0.26, 0.84]** |
| MDD | 0.01 [-0.04, 0.07] | **0.13 [0.08, 0.18]** | **0.14 [0.09, 0.19]** | **0.15 [0.10, 0.21]** |

**Table S4.** Effect sizes for the three cardio-metabolic conditions calculated separately in patients with a psychiatric disorder and controls free of a psychiatric disorder.

| **Cardio-Metabolic Disorder** | | **QRI Gray Matter Thickness** | **QRI Gray Matter Subcortical Volume** | **QRI White Matter** | **Whole-brain QRI** |
| --- | --- | --- | --- | --- | --- |
| ***SMI+ (N=1618)*** |  |  |  |  |  |
| High Cholesterol (N=325) | | 0.04 [-0.08, 0.16] | 0.05 [-0.07, 0.17] | 0.08 [-0.05, 0.20] | 0.09 [-0.03, 0.21] |
| Diabetes (N=94) | | 0.20 [-0.01, 0.41] | **0.25 [0.04, 0.46]** | **0.32 [0.11, 0.53]** | **0.41 [0.20, 0.62]** |
| Hypertension (N=492) | | 0.00 [-0.10, 0.11] | **0.12 [0.02, 0.23]** | **0.22 [0.12, 0.33]** | **0.19 [0.08, 0.29]** |
| ***SMI- (N=11849)*** |  |  |  |  |  |
| High Cholesterol (N=1790) | | -0.02 [-0.07, 0.03] | -0.01 [-0.06, 0.04] | 0.05 [0.00, 0.10] | 0.01 [-0.04, 0.06] |
| Diabetes (N=78) | | 0.03 [-0.19, 0.25] | -0.02 [-0.25, 0.20] | 0.20 [-0.02, 0.43] | 0.11 [-0.12, 0.33] |
| Hypertension (N=2841) | | 0.02 [-0.03, 0.06] | 0.03 [-0.01, 0.07] | **0.21 [0.17, 0.25]** | **0.14 [0.09, 0.18]** |

**Table S5.** ANOVA F and p-values for the effects of antipsychotic, Lithium, antidepressant, and mood stabilizing medications each on QRI and their interactive effects with metabolic disorders. **Bolded** values indicate significance after Bonferroni correction for N=4 comparisons (p<0.05/4=0.0125).

| ***Antipsychotic Medication*** | **QRI Gray Matter Thickness** | **QRI Gray Matter Subcortical Volume** | **QRI**  **White Matter** | **Whole-brain**  **QRI** |  |
| --- | --- | --- | --- | --- | --- |
| Antipsychotics | 0.02 | **9.23** | *5.20* | **6.99** |  |
|  | (0.89) | **(2.24×10^-3^)** | *(0.02)* | **(8.28×10^-3^)** |  |
| CMD | 0.05 | *4.56* | **10.45** | **7.06** |  |
|  | (0.82) | *(0.03)* | **(1.25×10^-3^)** | **(7.94×10^-3^)** |  |
| Antipsychotics*CMD | 0.77 | 0.40 | 1.68 | 2.08 |  |
|  | (0.38) | (0.53) | (0.20) | (0.15) |  |
| ***Lithium Medication*** |  |  |  |  |  |
| Lithium | 0.03 | 0.09 | 0.67 | 0.21 |  |
|  | (0.86) | (0.76) | (0.41) | (0.65) |  |
| CMD | 0.05 | *5.38* | **11.53** | **7.95** |  |
|  | (0.83) | *(0.02)* | **(7.00×10^-4^)** | **(4.86×10^-3^)** |  |
| Lithium*CMD | 0.02 | 0.003 | 1.27 | 0.18 |  |
|  | (0.88) | (0.95) | (0.26) | (0.67) |  |
| ***Antidepressant Medication*** |  |  |  |  |  |
| Antidepressants | 0.88 | 0.77 | 2.15 | 2.45 |  |
|  | (0.35) | (0.38) | (0.14) | (0.12) |  |
| CMD | 0.05 | *5.36* | **11.33** | **7.89** |  |
|  | (0.82) | *(0.02)* | **(7.81×10^-4^)** | **(5.02×10^-3^)** |  |
| Antidepressants*CMD | 0.05 | **6.30** | 0.04 | 1.69 |  |
|  | (0.82) | **(0.01)** | (0.85) | (0.19) |  |
| ***Mood Stabilizers*** |  |  |  |  |  |
| Mood Stabilizers | 0.0003 | 1.87 | 0.16 | 0.77 | |
|  | (0.99) | (0.17) | (0.69) | (0.38) | |
| CMD | 0.05 | *5.30* | **11.24** | **7.82** | |
|  | (0.82) | *(0.02)* | **(8.17×10^-4^)** | **(5.23×10^-3^)** | |
| Mood Stabilizers* CMD | 0.64 | 0.004 | 0.30 | 0.04 | |
|  | (0.42) | (0.95) | (0.58) | (0.85) | |

**Table S6.** Linear model results for antipsychotic and lithium medications on QRI and interactive effects with diabetes, hypertension, and high cholesterol.

**Bolded** values indicate significance after Bonferroni correction for N=4 comparisons (p<0.05/4=0.0125).

*Italicized* values indicate trend-level significance (0.0125<p<0.05)

| ***Antipsychotic Medication*** | **QRI Gray Matter Thickness** | **QRI Gray Matter Subcortical Volume** | **QRI White Matter** | **Whole-brain**  **QRI** |
| --- | --- | --- | --- | --- |
| Diabetes | **0.05±0.02** | 0.02±0.02 | *0.03±0.01* | **0.03±0.01** |
|  | **(3.07×10^-3^)** | (0.22) | *(0.02)* | (**5.08×10^-3^)** |
| Antipsychotic | -0.01±0.02 | 0.03±0.02 | 0.03±0.02 | 0.02±0.01 |
|  | (0.63) | (0.09) | (0.13) | 0.18 |
| Diabetes*Antipsychotic | 0.02±0.05 | **0.17±0.06** | 0.07±0.05 | **0.09±0.04** |
|  | (0.66) | **(2.72×10^-3^)** | (0.21) | **(1.09×10^-2^)** |
| Hypertension | 0.00005±0.01 | *0.02±0.01* | **0.03±0.01** | **0.01±0.004** |
|  | (0.94) | *(0.05)* | **(2.32×10^-4^)** | **(6.44×10^-3^)** |
| Antipsychotic | -0.01±0.02 | 0.04±0.02 | 0.02±0.02 | 0.02±0.01 |
|  | (0.82) | (0.08) | (0.40) | (0.23) |
| Hypertension*Antipsychotic | 0.01±0.04 | 0.03±0.04 | 0.04±0.03 | 0.03±0.02 |
|  | (0.85) | (0.38) | (0.21) | (0.22) |
| High Cholesterol | -0.002±0.01 | 0.003±0.01 | 0.01±0.01 | 0.002±0.01 |
|  | (0.78) | (0.78) | (0.51) | (0.80) |
| Antipsychotic | -0.01±0.02 | 0.04±0.02 | 0.02±0.02 | 0.01±0.01 |
|  | (0.52) | (0.11) | (0.42) | (0.36) |
| High Cholesterol*Antipsychotic | 0.04±0.04 | 0.06±0.04 | 0.07±0.04 | *0.06±0.03* |
|  | (0.33) | (0.12) | (0.07) | *(0.03)* |
| ***Lithium Medication*** |  |  |  |  |
| Diabetes | **0.05±0.01** | *0.03±0.02* | **0.04±0.01** | **0.04±0.01** |
|  | **(1.31×10^-3^)** | *(0.02)* | **(3.06×10^-3^)** | **(1.32×10^-4^)** |
| Lithium | 0.01±0.03 | -0.01±0.04 | -0.02±0.03 | -0.01±0.02 |
|  | (0.79) | (0.80) | (0.46) | (0.72) |
| Diabetes*Lithium | N/A | N/A | N/A | N/A |
| Hypertension | 0.001±0.01 | **0.02±0.01** | **0.03±0.01** | **0.02±0.005** |
|  | (0.89) | **(0.01)** | **(1.35×10^-4^** | **(2.16×10^-3^)** |
| Lithium | 0.01±0.04 | 0.03±0.04 | *-0.09±0.04* | -0.02±0.03 |
|  | (0.81) | (0.55) | *(0.02)* | (0.49) |
| Hypertension*Lithium | -0.01±0.07 | -0.12±0.08 | **0.21±0.07** | 0.03±0.05 |
|  | (0.87) | (0.13) | **(3.12×10^-3^)** | (0.58) |
| High Cholesterol | -0.001±0.01 | 0.01±0.01 | 0.01±0.01 | 0.01±0.01 |
|  | (0.91) | (0.47) | (0.08) | (0.27) |
| Lithium | -0.001±0.04 | -0.04±0.05 | 0.07±0.04 | 0.01±0.03 |
|  | (0.97) | (0.38) | (0.07) | (0.69) |
| High Cholesterol*Lithium | 0.02±0.07 | 0.07±0.07 | **-0.28±0.07** | -0.06±0.05 |
|  | (0.77) | (0.33) | **(4.73×10^-5^)** | (0.19) |

**Table S7**. Adjusted p-values of pairwise comparisons of groups of patients using antipsychotic, lithium, antidepressant, and mood stabilizing medication (AP+, LM+, AD+, MS+) and with cardio-metabolic disorders (CMD+).

**Bolded** values indicate significance.

| ***Antipsychotic Medication (AP)*** | **QRI Gray Matter Thickness** | **QRI Gray Matter Subcortical Volume** | **QRI White Matter** | **Whole-brain QRI** |
| --- | --- | --- | --- | --- |
| AP+~AP- | 0.89 | **0.002** | **0.02** | **0.008** |
| AP+/CMD-~AP-/CMD- | 0.88 | 0.44 | 0.97 | 0.92 |
| AP-/CMD+~AP-/CMD- | 1.00 | 0.20 | **0.02** | 0.09 |
| AP+/CMD+~AP-/CMD- | 0.96 | 0.008 | **0.005** | **0.003** |
| AP-/CMD+~AP+/CMD- | 0.88 | 0.77 | 0.99 | 1.00 |
| AP+/CMD+~AP+/CMD- | 0.80 | 0.72 | 0.22 | 0.21 |
| AP+/CMD+~AP-/CMD+ | 0.96 | **0.05** | 0.08 | **0.03** |
| ***Lithium Medication (LM)*** |  |  |  |  |
| LM+~LM- | 0.86 | 0.76 | 0.41 | 0.65 |
| LM+/CMD-~LM-/CMD- | 1.00 | 0.99 | 0.99 | 1.00 |
| LM-/CMD+~LM-/CMD- | 0.99 | 0.09 | **0.003** | **0.02** |
| LM+/CMD+~LM-/CMD- | 0.99 | 1.00 | 0.80 | 0.99 |
| LM-/CMD+~LM+/CMD- | 1.00 | 0.93 | 0.99 | 0.98 |
| LM+/CMD+~LM+/CMD- | 0.99 | 0.99 | 0.86 | 0.99 |
| LM+/CMD+~LM-/CMD+ | 0.99 | 0.99 | 0.46 | 0.88 |
| ***Antidepressant Medication (AD)*** | |  |  |  |
| AD+~AD- | 0.35 | 0.38 | 0.14 | 0.11 |
| AD+/CMD-~AD-/CMD- | 0.94 | 0.81 | 0.73 | 0.98 |
| AD-/CMD+~AD-/CMD- | 1.00 | 0.76 | **0.02** | 0.20 |
| AD+/CMD+~AD-/CMD- | 0.83 | **0.01** | **0.02** | **0.007** |
| AD-/CMD+~AD+/CMD- | 0.96 | 0.43 | 0.77 | 0.80 |
| AD+/CMD+~AD+/CMD- | 0.99 | **0.006** | 0.36 | 0.08 |
| AD+/CMD+~AD-/CMD+ | 0.87 | 0.06 | 0.69 | 0.19 |
| ***Mood Stabilizers (MS)*** |  |  |  |  |
| MS+~MS- | 0.99 | 0.17 | 0.69 | 0.38 |
| MS+/CMD-~MS-/CMD- | 0.96 | 0.69 | 1.00 | 0.85 |
| MS-/CMD+~MS-/CMD- | 1.00 | 0.11 | **0.004** | **0.03** |
| MS+/CMD+~MS-/CMD- | 0.91 | 0.99 | 0.99 | 0.99 |
| MS-/CMD+~MS+/CMD- | 0.96 | 0.31 | 0.79 | 0.39 |
| MS+/CMD+~MS+/CMD- | 0.84 | 0.96 | 1.00 | 0.89 |
| MS+/CMD+~MS-/CMD+ | 0.92 | 0.85 | 0.91 | 0.98 |
